# Thin Film Protected Flexible Nanoparticle Strain Sensors: Experiments and Modeling

**DOI:** 10.3390/s20092584

**Published:** 2020-05-01

**Authors:** Evangelos Aslanidis, Evangelos Skotadis, Evangelos Moutoulas, Dimitris Tsoukalas

**Affiliations:** 1 Department of Applied Physics, National Technical University of Athens, 15780 Athens, Greece; evskotad@central.ntua.gr (E.S.); e.moutoulas@soton.ac.uk (E.M.); dtsouk@central.ntua.gr (D.T.); 2 Centre for Electronics Frontiers Zepler, Institute for Photonics and Nanoelectronics, University of Southampton Highfield Campus, University Road, Building 53 (Mountbatten), Southampton SO17 1BJ, UK

**Keywords:** flexible sensors, nanoparticle sensors, tunneling model, endurance, atomic layer deposition, strain sensors, naked, solvent-free

## Abstract

In this work, the working performance of Platinum (Pt), solvent-free nanoparticle (NP)-based strain sensors made on a flexible substrate has been studied. First, a new model has been developed in order to explain sensor behaviour under strain in a more effective manner than what has been previously reported. The proposed model also highlights the difference between sensors based on solvent-free and solvent-based NPs. As a second step, the ability of atomic layer deposition (ALD) developed Al_2_O_3_ (alumina) thin films to act as protective coatings against humidity while in adverse conditions (i.e., variations in relative humidity and repeated mechanical stress) has been evaluated. Two different alumina thicknesses (5 and 11 nm) have been tested and their effect on protection against humidity is studied by monitoring sensor resistance. Even in the case of adverse working conditions and for increased mechanical strain (up to 1.2%), it is found that an alumina layer of 11 nm provides sufficient sensor protection, while the proposed model remains valid. This certifies the appropriateness of the proposed strain-sensing technology for demanding applications, such as e-skin and pressure or flow sensing, as well as the possibility of developing a comprehensive computational tool for NP-based devices.

## 1. Introduction

In recent years, the development of flexible electronics has attracted a lot of interest due to its applications in robotics [1,2], wearable electronics [3,4,5,6], health-monitoring of large structures [7,8,9,10], and many other areas. Particularly in the field of flexible electronics, strain sensors are of great importance in emerging technologies such as the internet of things. Over the last decade, many novel nanomaterials have been used in strain sensing applications such as carbon nanotubes [11,12,13], nanowires [5,14,15], MoS_2_ [16], graphene [17], and nanoparticles (NPs) [6,18,19,20,21,22].

Strain sensors based on NP films, in particular, have been of growing interest due to their increased sensitivity [19,20,21] when compared to existing metal strain sensors that incorporate thin film technology [23]. In addition, the low processing temperatures required in the case of NP-based strain sensing devices, render them fully compatible with flexible substrate technology [24]. NP strain sensors are finding new applications in new areas such as healthcare [1,4] and particularly in the development of electronic skin [4,15,25,26,27]. Their increased sensitivity to strain can be attributed to fundamental charge-transport mechanisms in NP assemblies. Such conductivity mechanisms are governed by the quantum tunneling effect which relates exponentially to inter-NP distance [28]. Many research groups focus their interest on increasing the sensitivity of NP-based sensors, and usually, this can be achieved by incorporating NP films with varying conductivities, so as to manipulate the charge transport mechanisms of the device [19,20,22,29]. Lee et al. [19] studied the combination of metallic and insulated NPs as sensitive materials, and thereby combining Au NPs, CdSe NPs, and nanocracks, a gauge factor of up to 5045 was achieved. In the field of bio-inspired sensing devices, cracks have often been employed, so as to radically increase the sensor’s sensitivity [19,30,31,32]; Han et al. [31], created a crack-based strain-sensor by depositing Au NPs on top of a cracked Polydimethylsiloxane (PDMS) substrate, obtaining a sensitivity of 5888. However, high sensitivities are also possible without cracks on the substrate. Shengbo et al. [5] combined Ag NPs and nanowires, achieving a sensitivity of up to 3766.

Strain sensors utilizing Pt NPs (fabricated on oxidized silicon substrates via the DC sputtering technique) have been investigated in the past by this group, demonstrating an increased sensitivity [33] while, more recently, we have also investigated the means to protect them against humidity [34]. Zheng et al. [35] have also manufactured nanoparticle-based flexible strain sensors using a DC sputtering technique, highlighting their superior performance against the semiconductor gauges.

In the current paper, we focus on the sensing properties of Pt NP sensors made on flexible polyimide substrates. First, we discuss the sensor strain response up to 1.2% strain. We present a physical model to explain the observed increase of the g-factor with increasing strain. The model is compared not only with our data, but also with other results reported in the literature. Xie et al. [36] manufactured Pd NPs-based strain sensors, using the sputtering technique and found that the g-factor is not a constant, but changes over the applied strain range. Lee et al. [37] also reported non-linear behaviour of their sensor based on silver NPs. Up to now, most attempts to model the behaviour of strain sensing devices made by either solvent-free or colloidal NPs are based on the physical model proposed by Herman et al. [28], by directly applying it to the experimental data set or by applying minor modifications [36]. This approach however does not take into account key aspects of straining flexible devices that employ solvent-free NPs. Contrary to a uniform elongation of solely inter-particle gaps, which existed prior to any strain (the case of cross-linked NPs), the straining solvent-free NP-based devices results in the formation and rise of multiple new gaps that contribute in the exponential rise of device resistance. The model proposed herein is able to explain experimental differences in terms of sensing performance observed between solvent-free (naked) NP strain sensors and cross-linked NP strain sensors [28], and constitutes a better fit to solvent-free NP strain sensing data.

This analysis is followed by a study of the sensor response in a humid environment that is known to influence the long-term behaviour of NP strain sensors [38]. Ketelsen et al. [39] have also reported on the endurance characteristics of strain gauges on flexible substrates, based on cross-linked gold NPs after performing a large number of strain/relaxation cycles, without, however, modifying the humidity environment during the tests. As proposed [40] using SAXS measurements, water molecules incorporated between nanoparticles result in a swelling of the NP film which increases the resistance of the nanoparticle network in a competitive way to the measured strain. It is, therefore, crucial to investigate the efficient protection of flexible strain sensors against humidity, as well as the effectiveness of the protection itself in increased strain (offered by the increased flexibility of the polyimide substrate). To that end, the sensor’s endurance in repeated mechanical stress has been evaluated in varying environmental conditions, i.e., R.H. Meanwhile, naked (no ALD coating) as well as alumina-coated strain sensors (alumina coatings of 5.5 and 11 nm in thickness) were submitted to “fatigue experiments” (multiple strain cycles: 1000 cycles of 0 to 1.2% strain) in order to evaluate possible alumina degradation. The ability of such coatings to retain their protective properties against humidity and strain/fatigue has been determined by monitoring device resistance in various case scenarios, while R.H. has been modified between two extreme values (10%–70%). Our model is also applied to alumina-coated and uncoated NP networks (both before and after the endurance experiments), indicating its universal appliance to solvent-free NP-based strain sensors as well as its validity, even after operating the sensors in adverse conditions. An optimized device, suitable for a wide range of demanding applications (e.g., e-skin etc.), was eventually produced while at the same time a novel physical model brought new insight to the physical properties of solvent-free NP-based strain sensors. The current results expand our previously reported work on silicon-based strain sensors, through the incorporation of flexible substrates and the investigation of device robustness along with sensitivity, compactness, cost, and power efficiency, as well as resiliency to changes in environmental conditions, signaling a major step towards device standardization and its integration in commercial applications.

## 2. Materials and Methods

**Figure 1:**
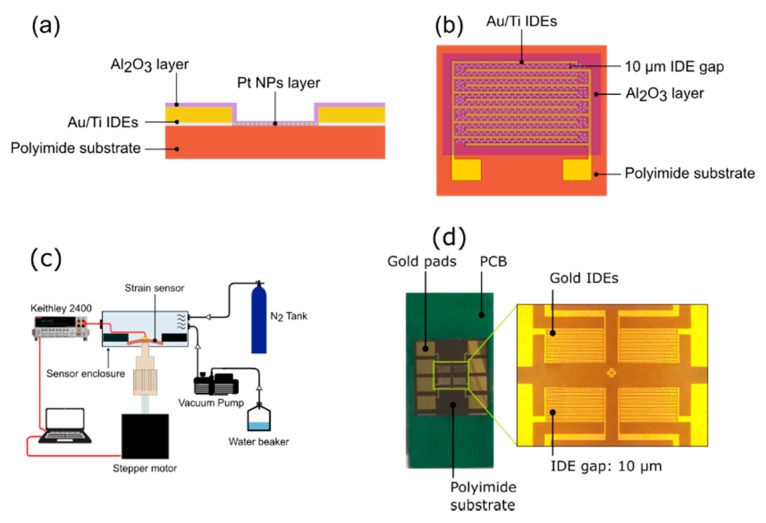
(**a**) Schematic cross section of the sensor (**b**) top down view of the sensor (**c**) Schematic of the experimental setup. (**d**) Image of the strain sensors on a PCB. There are 4 identical strain sensors in each device.

**Figure 2:**
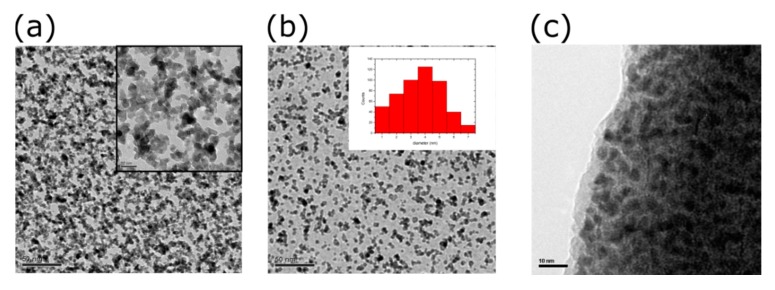
(**a**) TEM images of solvent-free Pt NPs with 50% surface coverage. Inset: higher magnification (**b**) TEM image of solvent-free Pt NPs with 28% surface coverage. Inset: Pt NPs size distribution (**c**) TEM image of Pt NPs covered with 5.5 nm thick alumina.

All fabrication experiments have been conducted at room temperature. All chemical reagents used in the current study have been supplied by Sigma Aldrich. Polyimide sheets with a mean thickness of 120 μm and a surface roughness of 0.7–0.8 nm, have been used as deposition substrates throughout the experiments. Prior to any processing, the polyimide substrates have been cleaned using IPA, DI water and ultra-sonication. Gold interdigitated electrodes (IDEs) have been patterned on top of polyimide substrates via optical lithography and the e-gun technique. At first, a thin Ti layer (approximately 4 nm) has been deposited using a deposition rate of 0.2 Å/sec, the Ti layer acts an adhesion layer between the gold and the polyimide substrate; as a second step a 30 nm gold layer has been deposited using a deposition rate of 0.5 Å/sec. As a final step, the lift-off technique has been used in order to produce the finalized IDEs structure. The overall height of the IDEs plays a critical role in the production of the sensors since it can “shadow” the polyimide substrate during the NP deposition step, thus preventing good contact between NPs and IDEs. The interdigitated electrodes (IDEs) inter-finger spacing (or electrode gap) was 10 μm, while their width was 2 μm (Figure 1a). Pt nanoparticles (Pt NPs), with a mean diameter of 4 nm and standard deviation of 1.5 nm (Figure 2b), were deposited on top of the IDEs using a modified dc magnetron sputtering system. Sputtering is a well-known room temperature technique for the production of both thin films and nanoparticles. Sputtering allows control over particle size, by adjusting the target material to the deposition substrate distance, over nanoparticle flux, by adjusting the argon flux, and nanoparticle density concentration on the substrate surface, by adjusting the deposition time. Both electrode and Pt NPs depositions were performed at 10^−5^ mbar pressure.

To investigate the protection of sensors against humidity, an alumina protective coating was deposited on top of the devices (Figure 1b) using an atomic layer deposition system (Picosun ALD R-200). The ALD deposited films are fabricated by consecutive cycles using specific precursors. The alumina precursors were tetramethylaluminum (TMA) and deionized water (DI water). During the deposition, the ALD reactor was under 10 mbar pressure and a constant flow of 300 sccm of 99.999% purity N_2_. The exposure time for both TMA and DI water was 0.1 s, while the purge time was 10 s for TMA and 15 s for DI water for each cycle. The deposition temperature was at 150 °C, for 50 and 100 cycles at each temperature, resulting in 5.5 nm and 11 nm of alumina respectively. This deposition temperature results in a low concentration of OH molecules in the film [34] that can facilitate the absorption of water molecules from the environment, while it is compatible with polyimide substrate temperature processing. Several fatigue tests were performed before and after the alumina deposition and the protective capabilities of the alumina coating from R.H. has been studied. The finalized strain-sensing devices have been characterized by electrical measurements, transmission electron microscopy (TEM) measurements, as well as optical microscopy measurements.

Sensor sensitivity was determined by resistance measurements, using a Keithley 2400 source meter, during the application of strain steps by a home-made experimental setup. Strain was applied with 0.007% precision, using a micrometric piston controlled by a stepper motor. The stepper motor is powered and controlled by a microcontroller (Arduino Uno) that allows the performance of reliable fatigue tests (small variations in the applied strain-range, for each strain cycle). The sensors were glued on a PCB board to ensure uniform deformation of the substrate during stress application (Figure 1d). The system was encased in a climate chamber in which R.H. and Temperature were controlled (Figure 1c). The relative humidity was controlled through the application of either Nitrogen, 99.999% in purity, or DI water vapors transported to the strain sensor’s chamber, from a tank using a mechanical vacuum pump. With this configuration, R.H. values between 10% and 70% were achieved. Both temperature and relative humidity were monitored by respective commercial sensors. Before any measurement, the stage was calibrated using a commercial flexible strain sensor with a gauge factor of 2.13. During the measurements, the temperature was kept constant at 23 °C, while the resistance was monitored by applying a constant voltage of 1 V.

## 3. Results and Discussion

### 3.1. Sensor Response Using Cross-Linked NPs

The operational principle of nanoparticle sensors is based on the fact that NPs have an inter-particle distance, which is key to the conductivity of the device. Assuming that the inter-particle distance is defined by *l*, the conductivity is given by the following equation:

(1) $$R=r_{0}exp\;\left(\beta l\right)\;exp\;\left(\frac{E_{c}}{K_{b}T}\right),$$

where *β* is the tunneling constant, $r_{0}$ a pre-exponential constant, $K_{b}$ is the Boltzmann constant, *T* the temperature, and $E_{c}$ the activation energy which is given by:

(2) $$E_{c}=\frac{e^{2}}{8\pi \varepsilon \varepsilon_{0}}\left(\frac{1}{r}-\frac{1}{r+l}\right),$$

where *r* is the mean diameter of the nanoparticles and *ε* the electric permittivity of the dielectric medium. At room temperature $E_{c}/K_{b}T\;\ll 1$. Hermman et al. [28] have proposed a theory suggesting that the differential resistance change is given by the following equation:

(3) $$\frac{\Delta R}{R}=\exp\left(g\gamma \right)-1$$

where *g* is the strain sensitivity or the strain gauge factor (g-factor). For small deformations *γ*, the Equation (3) becomes:

(4) $$\frac{\Delta R}{R}=g\gamma$$

**Figure 3:**
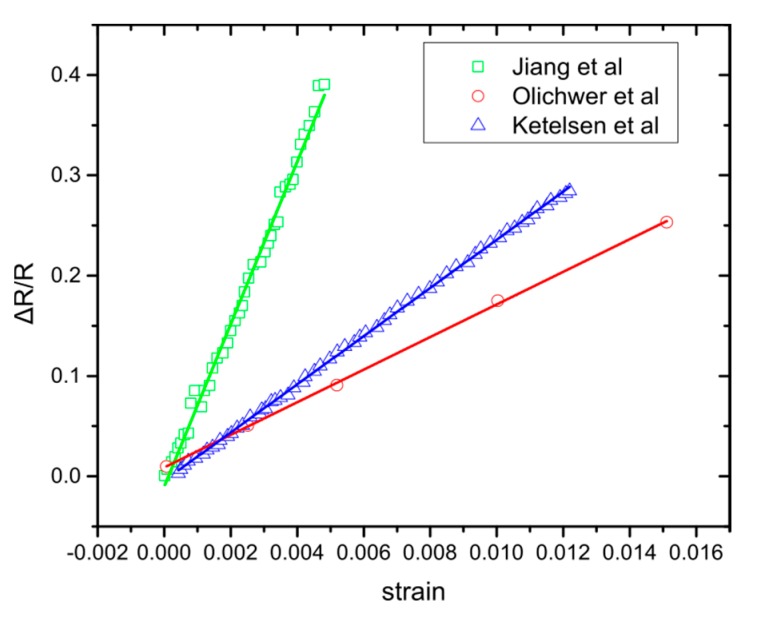
Relative resistance change over strain for different strain sensors based on cross-linked Gold nanoparticles Jiang et al. [41] Ketelsen et al. [39] and Olichwer et al. [42]. The continuous lines are the fitting of Equation (4).

This model precedes that the NPs are cross-linked and they all have an initial inter-particle gap *l*. When strain is applied, all the inter-particle gaps change from *l* to *l* + *dl*. All the cross-linked NPs have initial inter-particle gaps, which increase with the application of strain. The equation that describes the relative resistance change is Equation (3). In Figure 3, we use Equation (4) to compare with experimental results of strain NP sensors made on flexibles by using cross linked nanoparticles, as reported in the literature. The comparison shows that the above model is largely sufficient to describe the sensor response.

### 3.2. Sensor Response Using Solvent-Free NPs and Modeling

**Figure 4:**
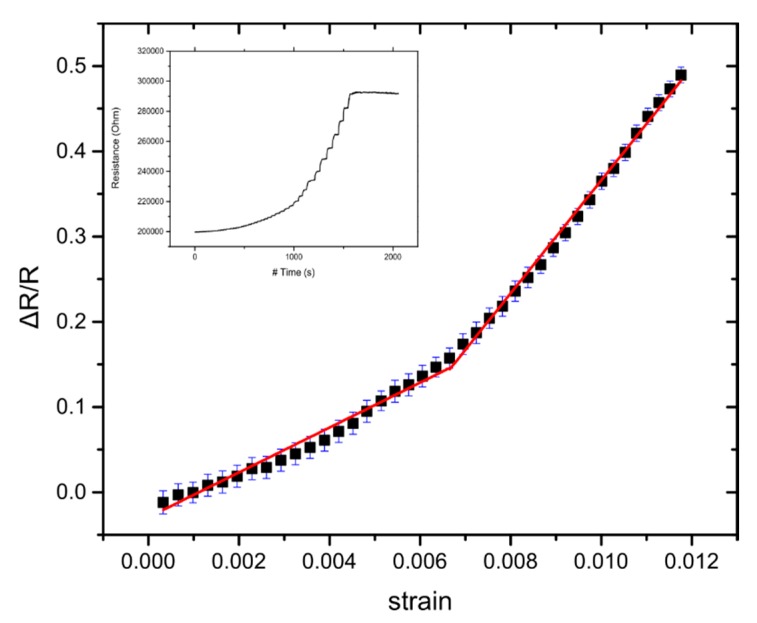
Measurement of resistance during gradual application of strain. Relative resistance over strain graph, where the GF is calculated by the slopes of the fitting line. Error bars represent the standard deviation of the measurements. In this particular example, the sensor has GF1~26 for lower strains and GF2~66 for higher strains.

The resistivity of the Pt NPs film formed in vacuum by sputtering and gas condensation strongly depends on the NP surface coverage. In previous work published by this group, the relation between NP surface coverage and sensor sensitivity has been investigated [33], concluding that the best-performing devices were achieved when the surface coverage is just below the percolation threshold (devices with a NP surface coverage of 50%) (Figure 2a). A typical response of Pt-NP sensors made on flexible substrates is shown in Figure 4.

**Figure 5:**
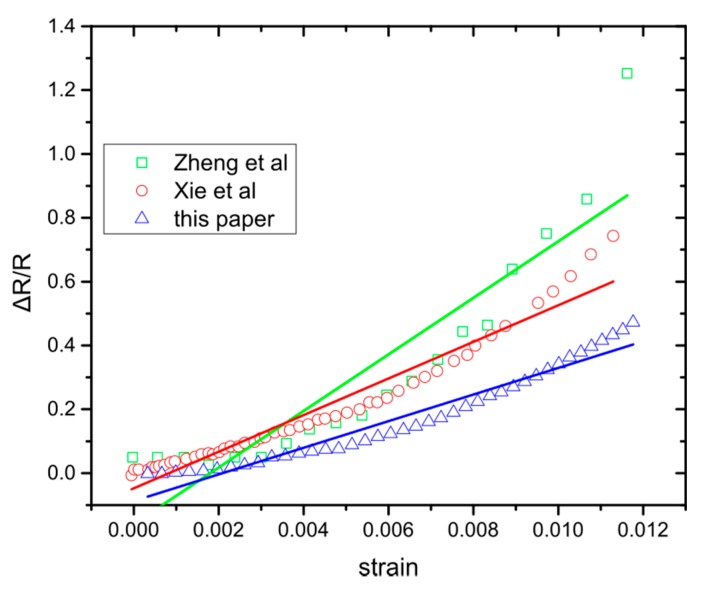
Relative resistance change over strain for different strain sensors based on sputtering deposited NPs. Zheng et al. [35] Cr NPs -based sensor, Xie et al. [36] Pd NPs -based sensor and this paper with Pt NPs. The colour lines are the fitting of Equation (4).

**Figure 6:**
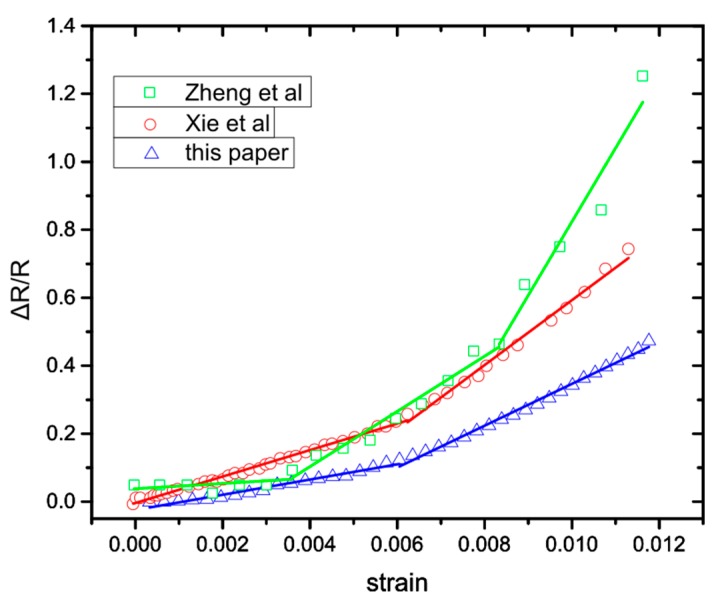
Relative resistance change over strain for different strain sensors based on sputtering deposited NPs. Zheng et al. [35] Cr NPs-based sensor, Xie et al. [36] Pd NPs -based sensor and this paper with Pt NPs. The solid-colour lines are the fitting of Equations (4) and (9) in the case of strain sensing devices, exhibiting two distinctive linear regions and the fitting of Equations (4), (9) and (12) for strain sensors with 3 different linear regions.

Figure 5 shows the relative resistance change of sputtered NPs on flexible substrates reported by different groups [35,36], underlying that Equation (3) is not able to describe their behaviour. The relative resistance change displays strain ranges that can be described with a linear Equation (4) and others where they are still linear but with a different slope (Figure 6) and, hence, different sensitivity. The existing model based on electron tunneling between NPs (Section 3.1) is perfectly adequate to describe the sensing performance of cross-linked NPs, but does not predict a change of the g factor value within the reported measurement range that still needs to be accounted for.

**Figure 7:**
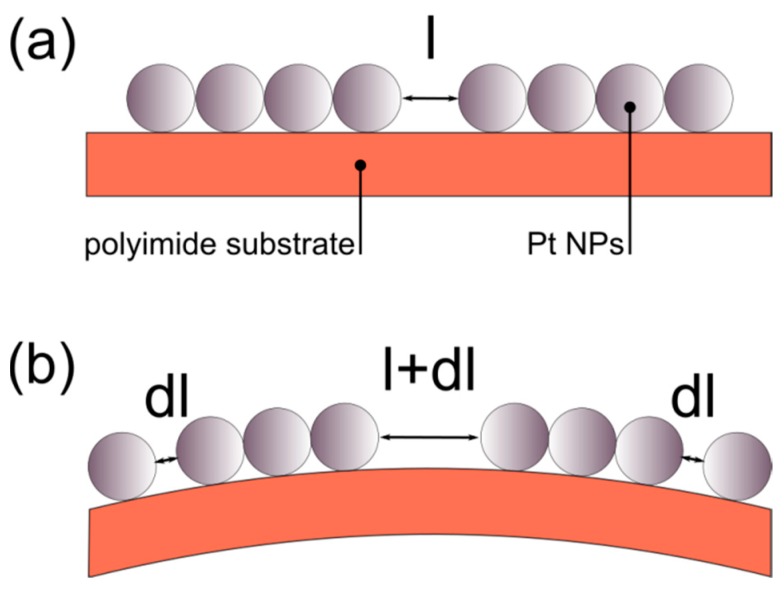
Graphic representation of inter-particle gaps between NP islands. At first there is a distance *l* (**a**) which after the appliance of strain is increased by dl, hence creating two additional gaps (**b**).

In our case, the Pt-NPs are not cross-linked; they soft-land on the substrate in what appears to be a random distribution, resulting in areas where all the NPs are in contact (therefore creating islands) and in other areas where an inter-particle distance exists between either islands or individual NPs. Therefore, when strain is applied, pre-existing inter-particle gaps increase, while larger NP islands fragment to smaller NP clusters (Figure 7). This clearly highlights the need for revisiting the model proposed in Section 3.1 and ultimately producing a new, appropriate model, as discussed below.

At room temperature, if the mean value of the initial inter-particle distance is *l*, the resistivity is given by:

(5) $$R_{0}=r_{0}\exp\left(\beta l\right)$$

By applying strain, it is possible to create some new inter-particle distances. Therefore, the resistivity will be given by:

(6) $$R_{s}=R_{0}\exp\left(\beta dl\right)+Nr_{0}\exp\left(\beta dl\right)$$

where the first term results from the pre-existing inter-particle gaps and the second from the new ones. *N* is a dimensionless number that depends on several parameters like the number of the new inter-particle gaps, as well as on how strongly they contribute to the overall resistivity. For example if all the NPs are assembled in a straight line, then all the inter-particle gaps will contribute the same. In our case however, where the NPs create complex paths with most likely several possible conductive pathways that are parallel to each other, each inter-particle gap contributes differently to the final resistance. In addition, N depends on the strain value that creates the new inter-particle gaps. If *N* = *kγ*, with k defined as the number of gaps/strain unit (this being valid above a threshold strain value) the differential resistance change is now given by:

$$\frac{\Delta R}{R_{0}}=\frac{R_{0}\exp\left(\beta dl\right)+k\gamma r_{0}\exp\left(\beta dl\right)-R_{0}}{R_{0}}$$

from which we obtain Equation (7):

(7) $$\frac{\Delta R}{R_{0}}=\exp\left(\beta dl\right)-1+\frac{k\gamma r_{0}\exp\left(\beta dl\right)}{R_{0}}$$

Since strain *γ* is defined by *γ* = *dl*/*l*, we can obtain that *dl* = *γl*. Introducing it to Equation (7) and considering that *g* = *βl*, we obtain:

(8) $$\frac{\Delta R}{R_{0}}=\exp\left(g\gamma \right)-1+\frac{k\gamma r_{0}}{R_{0}}\;\exp\left(g\gamma \right)$$

For small deformation, the equation becomes:

$$\frac{\Delta R}{R_{0}}=g\gamma +\frac{kr_{0}}{R_{0}}\gamma$$

From which we obtain Equation (9):

(9) $$\frac{\Delta R}{R_{0}}=\gamma \left(g+\frac{kr_{0}}{R_{0}}\right)$$

Within the parenthesis is the modified g-factor that is valid above a threshold strain value where new gaps start to form (and influence *R*) while *g* denotes the g factor below this strain threshold.

Assuming that, after the application of *nγ* strain, new inter-particle distances are created, the resistance will be given by:

(10) $$R_{s}=R_{0}\exp\left(\beta \left(ndl\right)\right)+k\gamma r_{0}\exp\left(\beta ndl\right)+k^{\prime }\gamma r_{0}\exp\left(\beta dl\right)$$

where *k*’ is the equivalent of *k* for the newest distances, and *n* the number of steps that strain *γ* was applied. Then, the differential resistance change will be given by:

(11) $$\frac{\Delta R}{R_{0}}=\exp\left(g\gamma \right)-1+\gamma \frac{k\gamma r_{0}}{R_{0}}\;\exp\left(g\gamma \right)+\frac{k^{\prime }\gamma r_{0}}{R_{0}}\;\exp\left(g\frac{\gamma }{n}\right)$$

For small deformation, this equation becomes:

(12) $$\frac{\Delta R}{R_{0}}=\gamma \left(g+\frac{kr_{0}}{R_{0}}+\frac{k^{\prime }r_{0}}{R_{0}}\right)$$

Again, inside the parenthesis is the new g-factor that depends on the strain value that creates the new inter-particle gaps. Equations (4), (9), and (12) are linear with different slopes and as can be seen in Figure 6, they fit more accurately the behaviour of the sensor than the previously reported model. Because of the “random” nature of the NP deposition, it is impossible to know explicitly the strain values for which new inter-particle gaps are formed, influencing the sensor response. For example in Figure 6 (green lines) we observe that three lines are required to fit the graph accurately, which suggests that a critical threshold of new inter-particle gaps has been reached twice, due to strain application. Each group was created at a different strain value, resulting in the gradual increase of the sensor’s response. If the NPs’ allocation was different, the change in the sensor’s response would be observed at a different strain value. In our experiments this happens around 0.64% strain. In addition, Equations (9) and (12) indicate why randomly deposited NPs should have different behaviour from cross-linked NPs. Finally, from Figure 5 and Figure 6, the Pearson’s correlation coefficient (Pearson’s r) has been calculated, after fitting Herrmann’s model (Figure 5) and the model proposed herein (Figure 6); the coefficient has a value between +1 and −1, where 1 stands for total positive linear correlation. The results (Table 1) indicate that the fitting lines generated from our model show better linear correlation than the ones deriving from Herrmann’s model.

### 3.3. Model Effectiveness and Exposure in Adverse Conditions

#### 3.3.1. Fatigue Experiments

**Figure 8:**
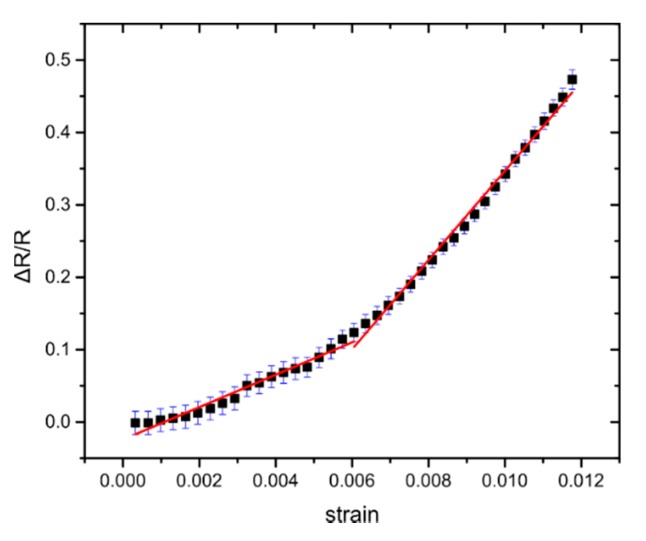
Relative resistance change over strain of a sensor after applying 1000 cycles of 1.2% strain. Error bars represent the standard deviation of the measurements. Our model predicts the sensor behaviour, using two linear slopes in order to fit the experiment results. The sensor has GF1~22 for lower strains and GF2~61 for higher strains.

In this section, we investigate the sensors’ performance under close to real-word operational conditions and then we compare the model with the experimental results, in order to test its validity. In all the results presented herein, NP surface coverage was close to 50% (established after transmission electron microscopy measurements using carbon grids) corresponding to a resistance value of hundreds of kΩs and to optimum device sensitivity. For the strain sensing experiments, 10 distinctive sensors have been employed. The sensors were submitted to fatigue tests where the GF was measured after the application of 1000 cycles of applied strain up to 1.2%. The value of the sensors’ G-factor has been determined from the slope of ΔR/R-strain graphs (Figure 4), where two G-factors were extracted from the graphs: one for small strain values (GF1 for γ < 0.64%) and one for large strain values (GF2 for γ > 0.64%). This behaviour is also in agreement with our model that predicts different GF for different strain. For all measurements, temperature and R.H. were kept constant and the mean value of the GFs remained practically unchanged, regardless of the number of strain cycles. Changes in the GF after 1000 cycles of fatigue tests can be seen in Supplementary Figure S1 (Mean GF1 of 19, and GF2 of 49 for the reference sensors and GF1 of 22 and GF2 of 45 for the sensors after fatigue tests). The effectiveness of the model to predict the strain sensing response of the solvent-free NP-based sensors can be seen in Figure 8, where even after 1000 strain cycles, the response of the sensors can be adequately fitted.

#### 3.3.2. Protection of the Sensor against Humidity and Model Effectiveness

Even though the GF of the devices remains stable after 1000 cycles of fatigue experiments and for constant R.H., unprotected Pt NP films remain sensitive to environmental changes, such as changes in R.H. Variations of R.H. influence the resistance of the device, therefore affecting its strain sensitivity. An unprotected device tends to be more sensitive and, furthermore, as discussed by Kano et al. [43], it has a linear response to humidity and could be therefore used as a humidity sensor. This effect leads to uncertainty in the value of the applied external stimulus and in an increase in the sensor’s strain detection limit. Preserving the high sensitivity of the NP strain sensors, without it being correlated to any R.H. variations, is crucial for their use. This is what a protective ALD deposited coating of Al_2_O_3_ has to accomplish. It is therefore crucial to investigate the validity of the proposed model in varying humidity conditions.

Firstly and in order to evaluate the degradation, if any, of the alumina coatings after intensive fatigue tests and for varying humidity environments, a total of 20 sensors were employed for the results discussed below. More specifically the sensors have been protected with two different alumina coatings, namely 5.5 or 11 nm, using the ALD technique. In Figure 2c, a TEM image of Pt NPs covered with a 5.5 nm thick alumina can be seen. It is worth noting that alumina films thicker than 5.5 nm resulted in images of poor quality due to the insulating properties of the alumina layer. Patsiouras et al. [34] have studied the protective properties of alumina coatings against relative humidity for strain sensors and found that the minimum alumina thickness required for adequate R.H. protection was 5.5 nm (deposited at 150 °C). However, this study was performed with NP strain sensors made on silicon substrates and, consequently, the strain values applied were low. In the case of flexible substrates studied herein, a 5.5 nm thick alumina coating failed to protect the sensor from humidity. We anticipate that a probable explanation for that is the formation of cracks in the alumina layer at high strain values, which create free paths for water molecules to penetrate the film. For that reason, alumina films of 11 nm in thickness have been employed in all of the following results.

**Figure 9:**
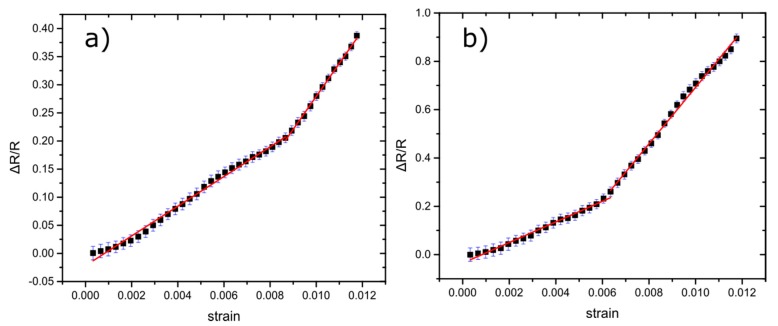
(**a**) Relative resistance change over strain graph for a sensor right after the alumina deposition. Error bars represent the standard deviation of the measurements. Two straight lines were used to fit, accurately, the experimental results as our model has proposed. In this example, the sensor has GF1~20 for lower strains and GF2~50 for higher strains. (**b**) Relative resistance change over strain graph for a sensor with alumina coating after 1000 stress cycles up to 1.2% strain. The sensor has GF1~60 for lower strains and GF2~85 for higher strains.

Before examining the protective properties of alumina films against variations in R.H, the effect of the alumina layer itself on sensor performance has been evaluated in parallel with the efficacy of the model proposed in Section 3.2. Figure 9a shows the performance of an alumina-protected strain sensor right after the alumina deposition and as can be seen the GF of the device has been reduced by 33% (Supplementary Figure S1). This happens probably due to the alumina’s built-in stress, which hinders, to some extent, NP dislocation [34]. The need of a two straight-line model in order to accurately fit the experimental results is also evident. We can also observe that the second line starts from a higher strain value than usual (~0.9% instead of 0.64%, which is the typical strain value for the second line). For high strain values (0.9%), the film starts to relax (in terms of stress) and the NPs start to move more freely. In Figure 9b, the performance of an alumina-coated sensor after 1000 stress cycles can be seen; the sensor is much more sensitive compared to uncoated sensors as well as to alumina-coated sensors that have not been subjected to fatigue tests. The fatigue experiments induce cracks in the alumina film which enhance NP dislocation in the vicinity of the crack, resulting in much higher GF, as already reported in the literature [39]. The foretold argument is supported by the fact that the initial resistance of the alumina-coated sensors is also slightly increased by 3.7% after 1000 stress cycles. Ketelsen et al. [39] who performed similar fatigue tests on cross-linked gold NP sensors reported that after 1000 stress cycles a 5% increase of the resistance was observed, but afterwards the sensor performance remained unaltered for up to 10,000 cycles. They attribute this behaviour to the formation of microcracks within the cross-linked NP film. In our experiments, we can attribute this change to cracks formed in the alumina layer since NPs are fabricated using the sputtering technique, forming a two-dimensional network of non-cross-linked objects. Furthermore, our model fits accurately the experimental data, highlighting the need for a two-line model in order to achieve correct fitting.

**Figure 10:**
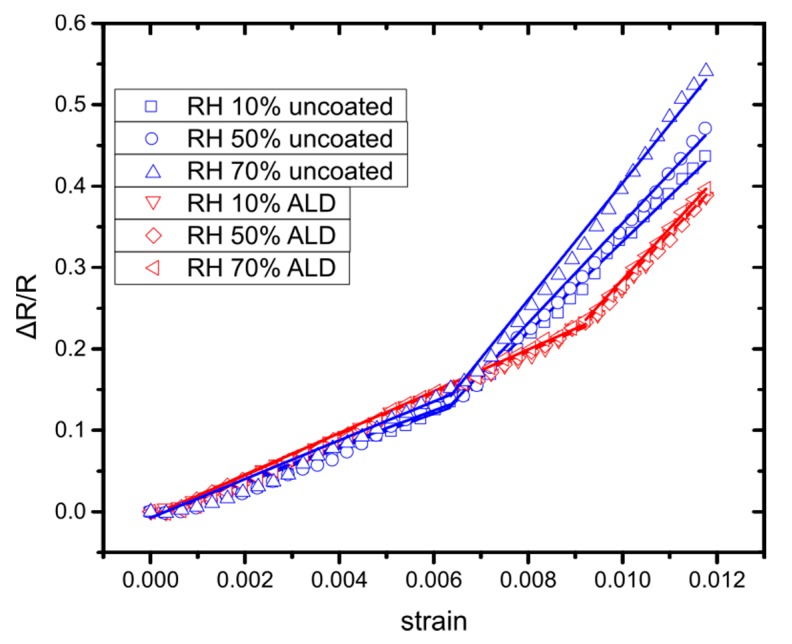
Performance of a sensor before alumina coating (uncoated) and right after the alumina coating (ALD) for RH conditions of 10%, 50% and 70%. The fitting lines for the uncoated sensor are the blue ones, while the red ones indicate the coated one.

As it has been reported [44,45], mechanical strain changes the sensor’s sensitivity towards R.H.; to that end, the protective properties of the 11 nm thick alumina layer against humidity were investigated by measuring the ΔR/R0 of the sensors under different R.H. conditions for unstrained sensors, as well as for strain values up to 1.2%. For each applied strain condition (0, 0.3, 0.6, 0.9 and 1.2%), we have varied the humidity from 10–70% and measured relative changes in resistance (Figure 10). It is worth mentioning that our model applies to all RH conditions (Figure 10) where two GFs are needed in order to fit the experimental data. The results in Figure 10 (and Supplementary Figure S2) indicate that in the case of uncoated sensors, ΔR/R0 changes attributed to R.H variation are comparable to resistance changes due to strain, therefore limiting the sensor performance. By using the alumina coating on top of the NPs, the effect of humidity is reduced below 2%, proving that the alumina coating is indeed an effective protective barrier against humidity [43]. This results in a much increased sensor sensitivity, i.e., the protected NP sensors can detect strains down to 0.007% while the uncoated ones have a detection limit of 0.107% to strain. Fatigue tests were performed for 1000 strain cycles and for strains up to 1.2% with ΔR/R0 measured under different R.H. conditions (10–70%) for coated and uncoated sensors (Supplementary Figure S2). After 1000 strain cycles, the ΔR/R0 has been increased to 3% and for high strain values to 7.5%. In any case, it remained much lower than the one measured for samples without the protective alumina coating. Finally, it is worth mentioning that an alumina coating of 11 nm contributes towards enhanced sensor stability and performance over time. Measurements conducted one and three months following the initial experiments revealed that sensors with an alumina coating of 11 nm featured minimal variance in device resistance and device sensitivity (compared to uncoated sensors and sensors with a 5 nm coating, see Supplementary Figure S3).

## 4. Conclusions

We have developed an original physical model to account for the enhanced g-factor observed in solvent-free NP strain sensors made on flexible substrates and underlined its difference with the existing model. The previously reported model for electronic conduction in these films, which is based on electron tunneling between NPs, is adequate to describe cross-linked NP strain sensors but needed to be revisited and modified in order to fit and explain the behaviour of solvent-free NP devices. The striking difference arose from the fact that cross-linked NPs form a uniformly 2-D interconnected network just after deposition, while solvent-free NPs are randomly deposited. As a result, for higher strains, new gaps between NPs are created giving rise to the increased g-factor observed. The model proposed herein takes into account the properties of solvent-free NPs, creating an appropriate tool for the respective strain-sensing devices while offering significant insight into their physical properties.

As a next step a technical study of solvent-free based strain sensors was performed by investigating protective coatings against R.H., their limitations, and the eventual optimization of the device. To do so, we have investigated the environmental stability of solvent-free NP sensors as well as the effectiveness of the proposed model to fit experimental data, by exposing the sensors to adverse conditions. Alumina deposited via the ALD technique was used as the sole protective coating against humidity. The effect of fatigue on the protective qualities of the alumina layer has been evaluated under varying relative humidity conditions for both unstrained as well as for devices under applied strain; resistance variance of “solvent-free” (alumina-free) and alumina-coated devices, in varying relative humidity conditions and before any fatigue experiments, has been compared to their resistance variance after the fatigue experiments.

Our results associate alumina thickness with device endurance for varying humidity concentrations and stability over long periods of time. Such results prove that an alumina film thickness of 11 nm fabricated at 150 °C can effectively protect flexible NP-based strain sensors from humidity, even after repeated device-bending. The development of processes for effective device protection in close to real-life conditions validates the appropriateness of the proposed strain sensors for a wide range of demanding applications, such as e-skin, micro and nano electromechanical sensors, and pressure or flow sensors. Investigating the optimization of device sensitivity by combining alumina thin films and metallic nanoparticles and the development of a computational tool for predicting device properties are only some of the future prospects deriving from the current study.

## Figures and Tables

**Table 1:** Comparison between this paper and two strain-sensing devices based on solvent-free NPs

	Materials	Substrate	Fabrication Method	Herrmann’s Model Pearson’s r	This Paper’s Model Pearson’s r		
					First Line	Second Line	Third Line
Zheng et al. [35]	Cr NPs	PET	sputtering	0.91238	0.99059	0.96269	0.92329
Xie et al. [36]	Pd NPs	PET	sputtering	0.9711	0.99548	0.99491	-
this paper	Pt NPs	polyimide	sputtering	0.97147	0.98129	0.99651	-

## Supplementary Materials

The following are available online at https://www.mdpi.com/1424-8220/20/9/2584/s1, Figure S1: Variation in the Sensors’ GFs for uncoated as well as alumina coated sensors, before and after Fatigue experiments. Figure S2: Resistance variance for sensors under fixed strain (strain values of 0%, 0.3%, 0.6%, 0.9% and 1.2%), in a varying humidity environment. R.H. varies between 10% and 70%. Figure S3: Relative resistance change over time. Sensors’ resistance was measured after one day, one month and three months for sensors without alumina coating (uncoated) and with 5 nm and 11 nm alumina coating.
