# Supplementary material for: Thin Film Protected Flexible Nanoparticle Strain Sensors: Experiments and Modeling
Source: Sensors (Basel). 2020 May 1;20(9):2584. doi: 10.3390/s20092584 (PMC7248731; doi:10.3390/s20092584)
Supplement: Supplementary file 1 [file sensors-20-02584-s001.pdf]

Supplementary Materials

Thin Film Protected Flexible Nanoparticle Strain Sensors: Experiments and Modeling

Evangelos Aslanidis [1,*], Evangelos Skotadis [1], Evangelos Moutoulas [1,2] and Dimitris Tsoukalas [1]

[1] Department of Applied Physics, National Technical University of Athens, 15780 Athens, Greece; evskotad@central.ntua.gr (E.S.); e.moutoulas@soton.ac.uk (E.M.); dtsouk@central.ntua.gr (D.T.)
[2] Centre for Electronics Frontiers Zepler, Institute for Photonics and Nanoelectronics, University of Southampton Highfield Campus, University Road, Building 53 (Mountbatten), Southampton SO17 1BJ, UK
* Correspondence: evaaslani@central.ntua.gr; Tel.: +30-210-772-1679

Sensors performance

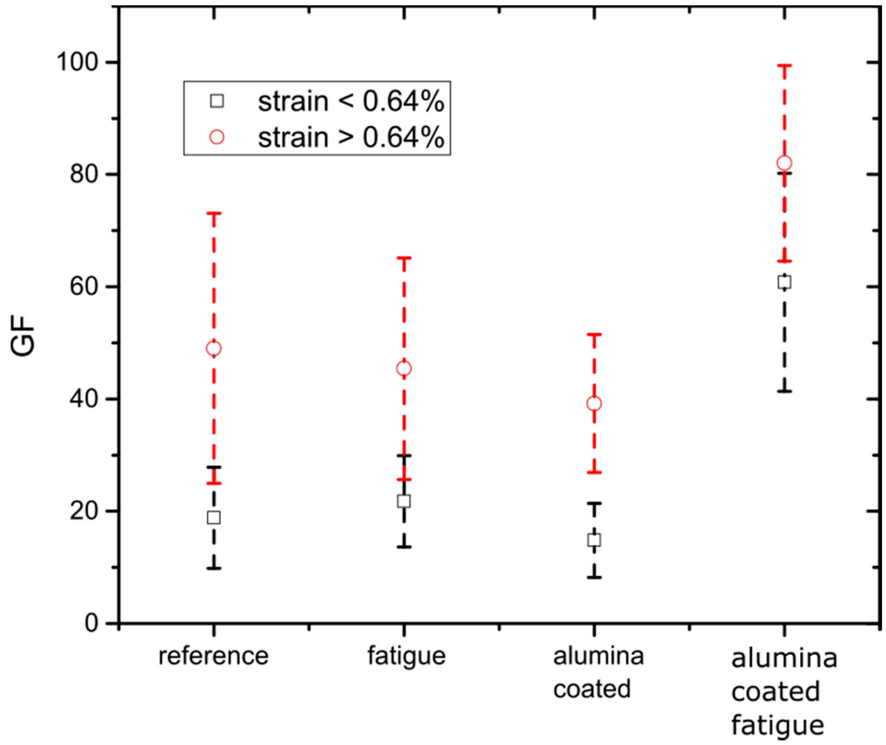

Figure S1. Variation in the Sensors' GFs for uncoated as well as alumina coated sensors, before and after Fatigue experiments.

The GF of all fabricated sensors has been measured for small ($\varepsilon < 0.64\%$) and large ($\varepsilon > 0.64\%$) strain values. The GFs of the uncoated sensors were measured right after the fabrication (GF1 = 19 with standard deviation (SD) of 9 and GF2 = 49 with SD of 24) and after 1000 cycles of stress up to 1.2% (GF1 = 22 with SD of 8 and GF2 = 45 with SD of 19). The GFs of the alumina coated sensors were also measured right after the alumina deposition (GF1 = 15 with SD of 6 and GF2 of 39 with SD of 12) and after 1000 cycles of stress up to 1.2 (GF1 = 61 with SD of 19 and GF2 = 82 with SD of 17) as can be seen in Figure S1. According to the results the fatigue test did not significantly affect the GFs of the uncoated sensors. After the alumina coating the GF was reduced by 33% for $\varepsilon < 0.64\%$ and by 13% for $\varepsilon > 0.64\%$. This reduction is attributed to the built in stress of the alumina film. After 1000 strain cycles (maximum strain up to 1.2%) for the alumina coated sensors, the mean GF value was increased by 77% for the small strain values and by 110% for large strain values; this is probably due to the

formation of cracks in the alumina film which in turn cause increased NP dislocation as well as increased GFs.

In Figure S2, Resistance changes for R.H. concentrations in the range of 10%–70% can be seen. Results correspond to sensors measured right after the fabrication (reference), after 1000 stress cycles up to 1.2% (fatigue), right after the alumina coating (alumina coated) and after 1000 stress cycles up to 1.2% (alumina coated fatigue). The measurements are performed by imposing a constant strain of 0, 0.3, 0.6, 0.9 or 1.2%. For each of the previous strain values (0%, 0.3%, 0.6%, 0.9% and 1.2%) R.H. varied between 10 and 70% while the resistance response was measured (without taking into account the resistance response to strain). Results in Figure S2 show the protective properties of the alumina film against R.H. The deferential resistance change for the uncoated sensors (both reference and fatigue) was between 11% and 15%, while for the alumina coated sensors the deferential resistance change was in the range of 0.5% to 2%. After the fatigue experiments the deferential resistance change increased to values between 3% and 7.5%.

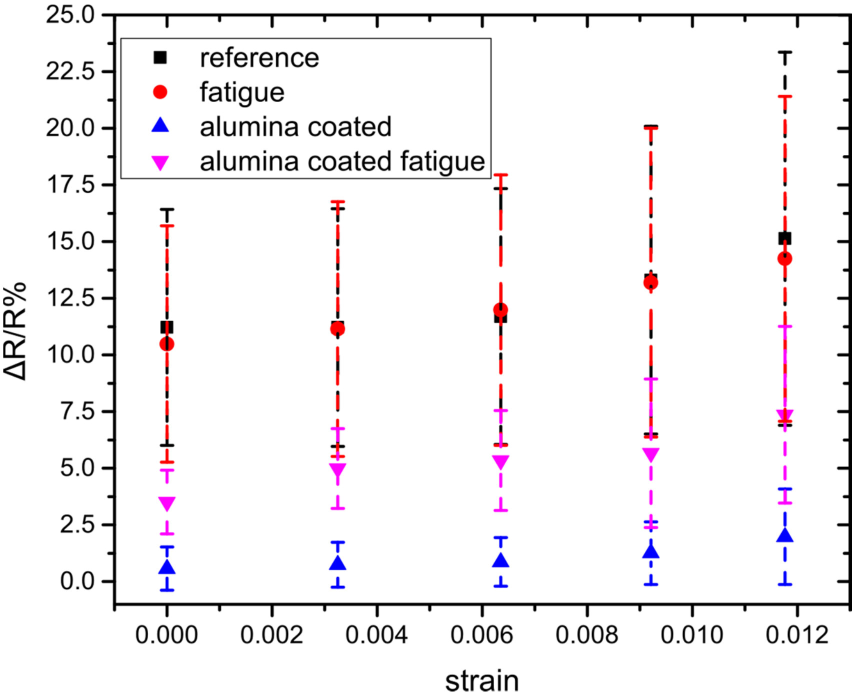

Figure S2. Resistance variance for sensors under fixed strain (strain values of 0%, 0.3%, 0.6%, 0.9% and 1.2%), in a varying humidity environment. R.H. varies between 10% and 70%.

Sensors stability and repeatability

After the fatigue experiments, each sensor was measured twice to ensure the reliability of the measurement. The sensors showed excellent repeatability, with minimal change in their GF (in the order of 0.5% for uncoated sensors and 0.2% for the coated ones). In addition, sensors' resistance was measured after one day, one month and three months to determine their endurance over time. As has been discussed, sensors GF is directly connected to the initial value of the resistance. All sensors were measured after 1000 cycles of strain up to 1.2%. Figure S3 shows the mean values of 30 sensors in total, 10 uncoated, 10 with 5nm alumina coating and 10 with 11 nm alumina coating. Error bars represent the standard deviation. For all sensors, the relative resistance change after a day was almost negligible. In particular, the uncoated sensors had a mean value of 0.37 with SD 0.1, the sensors with a 5 nm alumina coating a mean value of 0.018 with SD 0.08 and the sensors with the 11 nm alumina coating a mean value of 0.015 with SD 0.03. After one month uncoated sensors increased their resistance by 12% (SD of 2.1); after 3 months 6 out of 10 sensors increased their resistance by 59% (SD of 5.6) while 4 sensors were not operational. The GF of the operational sensors changed by 10% and 40% after 1 month and 3 months respectively. For 5 nm alumina coated sensors the increase was 6%

(SD of 2.4) after one month; after 3 months 7 out of 10 sensors increased their resistance by 21% (SD of 4.3) while 3 sensors rendered useless. The GF of the operational sensors changed by 5% and 15% after 1 month and 3 months respectively. Finally, the 11 nm alumina coated sensors featured an increased resistance by 1% (SD of 0.8) after one month, with 9 out of 10 sensors still remaining operational. After three months their resistance increased by 4% (SD of 1.4) with the same sensors still operating; the GF of the operational sensors changed by 1% and 2% after 1 month and 3 months respectively. The change in resistance led to a change in GF, making many of the uncoated sensors useless, while the majority of the protected sensors remained functional. Sensors with a 5 nm alumina coating featured again a significant change in resistance (especially after three months), indicating insufficient protection against humidity. On the other hand, an alumina coating of 11 nm in thickness prevented large resistance fluctuations, prolonging the operational lifespan of the sensors. The above results confirm the need for an alumina protective film, not only as a protection against humidity but also in order to extend the sensors' lifetime.

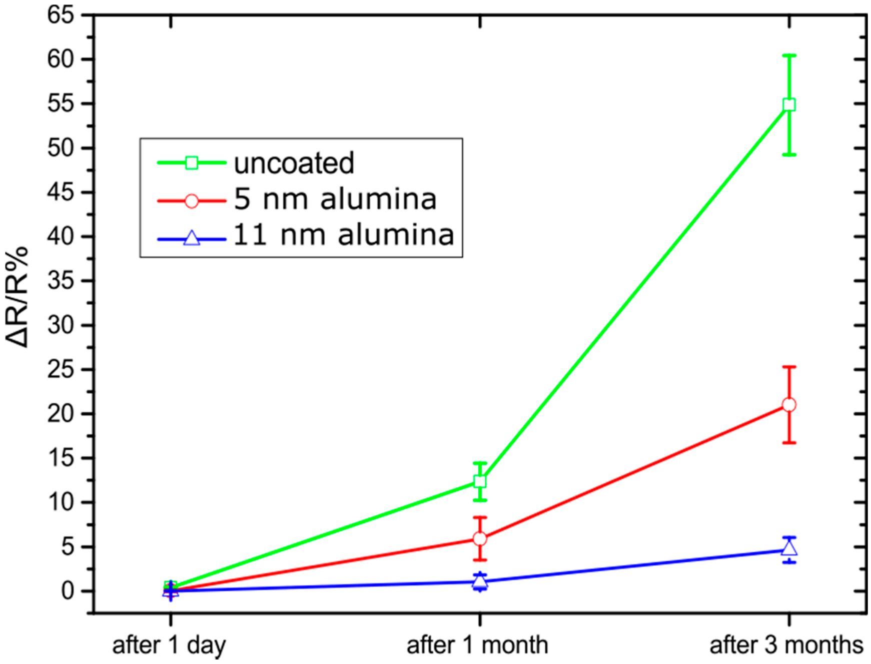

Figure S3. Relative resistance change over time. Sensors' resistance was measured after one day, one month and three months for sensors without alumina coating (uncoated) and with 5 nm and 11 nm alumina coating.
